# Efficient Utilization Method of Motorway Lanes Based on YOLO-LSTM Model

**DOI:** 10.3390/s25216699

**Published:** 2025-11-02

**Authors:** Xing Tong, Anxiang Huang, Yunxiao Pan, Yiwen Chen, Meng Zhou, Mengfei Liu, Yaohua Hu

**Affiliations:** College of Optical, Mechanical and Electrical Engineering, Zhejiang A&F University, Hangzhou 311300, China; 2023612021019@stu.zafu.edu.cn (X.T.); 2023612021007@stu.zafu.edu.cn (A.H.); 2024612021023@stu.zafu.edu.cn (Y.P.); 2023612021002@stu.zafu.edu.cn (Y.C.); 2024612021032@stu.zafu.edu.cn (M.Z.); 2024612021018@stu.zafu.edu.cn (M.L.)

**Keywords:** congestion warning, emergency lane using, Traffic Performance Index (TPI), You Only Look Once (YOLO), Long Short-Term Memory (LSTM)

## Abstract

With the development of cities, traffic congestion has become a common problem, which seriously affects the efficiency of motorway transport. This study proposed an improved ML-YOLO video data extraction model based on You Only Look Once (YOLOv8n) combined with the Deep Simple Online and real-time tracking (DeepSORT) algorithm, to classify the obtained Traffic Performance Index (TPI) into different congestion levels by extracting traffic flow parameters in real-time and combining with the K-means clustering algorithm. The Long Short-Term Memory Dropout (LSTM-Dropout) model and the emergency lane opening model were used to implement the road congestion warning successfully. The practicality and stability of the model were also verified by calculating the relative error between the predicted traffic flow parameters and the extracted parameters through the LSTM time series model. According to the model results, emergency lanes are opened when the motorway traffic TPI exceeds 0.17 and closed when below 0.17. This study provided a reasonable theoretical basis for motorway traffic managers to decide whether or not to open the emergency lane, effectively relieved motorway road congestion, improved efficiency of road traffic, and had important practical value and significance in reality.

## 1. Introduction

Motorway traffic congestion has become an increasingly severe issue with rapid urbanization, rising vehicle numbers, and insufficient infrastructure. Congestion reduces transport efficiency, increases emissions, and affects safety and quality of life. Solving the problem of traffic congestion on expressways has become a priority in urban research and planning.

Policy studies emphasize both direct and indirect strategies. Albalate and Fageda (2019) found that promoting public transport and regulating parking reduced congestion and accidents in Spanish cities [1], while Chow et al. (2014) showed that about 15% of London congestion was due to nonrecurrent factors such as accidents, stressing the need for dynamic solutions [2]. Metz (2018) reported that congestion charging in London and Stockholm yielded temporary benefits, whereas car ownership costs were more decisive in Singapore [3]. Zhu et al. (2021) highlighted how Intelligent Transportation Systems (ITSs) could relieve congestion in Jinan, China, where growth outpaced infrastructure [4]. Fosgerau and De Palma (2013) proposed time-varying parking fees to balance commuting flows [5], and Wang et al. (2015) used GPS taxi data to confirm daily predictability of traffic [6]. Koźlak and Wach (2018) emphasized demand-side factors such as business activity and car ownership in Polish cities, stressing the need for transport demand management [7].

Technological advances have created new opportunities. Traditional video monitoring was costly and subjective, but Tariq Abdullah (2020) showed that GPU-based cloud frameworks enable large-scale video analysis with up to 14× faster processing [8]. The Traffic Performance Index (TPI) has become a unified metric for congestion assessment. Zang et al. (2023) applied Self-Organizing Map (SOM) clustering to analyze Beijing’s patterns during COVID-19 [9]; He et al. (2016) developed segment-specific congestion indices [10]; Tu et al. (2022) used big data mining to refine flow predictions [11]; and Bao (2019) introduced multi-index fusion clustering for state identification [12]. Collectively, these studies underline TPI’s versatility for congestion monitoring, prediction, and classification.

Meanwhile, computer vision has transformed traffic management. Early work such as Tao et al. (2017) applied You Only Look Once (YOLO) for vehicle counting and improved detection in low light [13], while Mittal et al. (2023) integrated Faster R-CNN with YOLO for density estimation [14]. Beyond algorithmic advances, practical frameworks confirm YOLO’s potential: Jia-Ping Lin (2019) proposed a vehicle counting system with checkpoints [15]; Mohammed A. A. Al-qaness (2021) developed a YOLOv3-based surveillance system [16]; and Akhil Reddy Kalva (2021) combined YOLO with OpenCV for real-time tracking and classification [17].

More recent studies extend these capabilities. Samprit Bose (2021) showed that YOLOv3 could maintain >87% accuracy even at night [18]. Menghui Guo (2023) integrated YOLOv8 with Deep Simple Online and real-time tracking (DeepSORT) for dynamic vehicle counting and adaptive congestion analysis [19], while B. Sathiyaprasad (2025) introduced TrackNCount, which combines YOLOv8 and DeepSORT for real-time tracking, counting, and speed estimation across diverse scenarios [20]. These advances illustrate the shift from YOLOv3 to YOLOv8 and the growing role of combined detection–tracking systems in ITS.

In parallel, deep learning models for prediction, especially Long Short-Term Memory (LSTM), have become central to traffic forecasting. Zhao et al. (2017) captured spatiotemporal features with LSTM [21]; Ma et al. (2020) extended this to multi-lane convolutional LSTM [22]; and Lu et al. (2021) improved accuracy with temporal-aware modules [23]. Abduljabbar, Dia, and Tsai (2021) demonstrated the superiority of BiLSTM for speed forecasting and further validated the effectiveness of LSTM using real-time sensor data [24,25], while Dalgkitsis et al. (2018) designed cellular traffic predictors [26]. Oliveira et al. (2021) compared Multilayer Perceptron (MLP) and LSTM, finding MLP slightly better but confirming LSTM’s strength in sequential data [27]. Bogaerts et al. (2020) combined graph convolution with LSTM for multi-step forecasting [28], and Awan et al. (2020) enhanced prediction by integrating air pollution and environmental data [29]. Together, these works highlight the versatility of LSTM for congestion prediction. Building on these foundations, this study develops an ML-YOLO-based traffic congestion warning system with the following goals:

(1) To design an advanced real-time video-based model for extracting traffic flow parameters such as vehicle flow, speed, and density;
(2) To classify motorway congestion levels with machine learning and time series forecasting;
(3) To establish an emergency lane control strategy based on Traffic Performance Index (TPI) thresholds.

In this study, we tackle the ongoing challenge of urban traffic congestion by proposing an enhanced ML-YOLO model integrated with the DeepSORT algorithm for real-time tracking and congestion classification. This model, which leverages MobileViT and LSKb block modules within the YOLOv8n architecture, offers high predictive accuracy across key traffic parameters such as flow, density, speed, and the TPI, providing a robust framework for traffic monitoring and management. The system classifies congestion into distinct levels, enabling targeted interventions based on real-time data analysis. Additionally, the study introduces an LSTM-Dropout model combined with an emergency lane management system that dynamically adjusts based on congestion thresholds, optimizing traffic flow during peak times. The proposed system demonstrates the potential for smart traffic management and sets the foundation for future urban mobility solutions.

## 2. Materials and Methods

This study presents an end-to-end framework for video sensing, assessment, forecasting, and control on the motorway, designed for congestion warning and emergency lane management. The pipeline proceeds as follows: video acquisition and preprocessing; an improved ML-YOLO based on You Only Look Once (YOLOv8n) with a MobileViT Backbone and a Laplacian Smoothing Kernel (LSK) module for multi-class vehicle detection; Deep Simple Online and real-time tracking (DeepSORT) for cross-frame association to form stable trajectories; homography-based calibration to derive the three fundamentals of traffic flow—flow Q, speed V, and density K—from trajectories; construction of a Traffic Performance Index (TPI) and congestion stratification via K-means; short-term forecasting with a bi-directional LSTM-Dropout model; and a hysteresis-aware TPI threshold policy that triggers emergency lane openings and closings.

**Figure 1:**
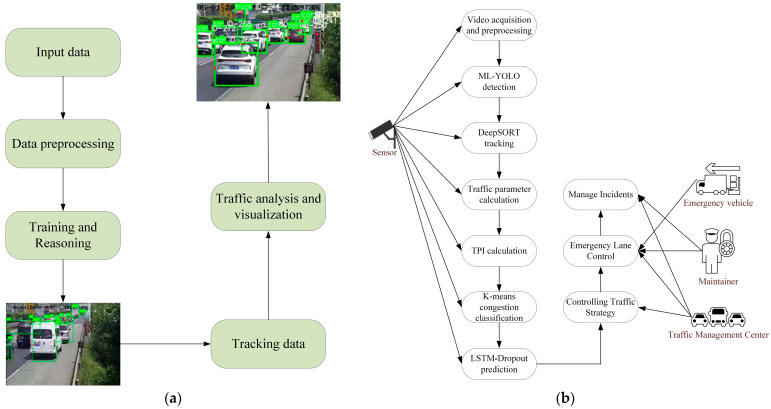
End-to-end pipeline and use-case architecture for video-based traffic analytics and emergency lane control. (**a**) Outline of the High-Level Process. (**b**) Interaction Between Video Surveillance Sensors and the Management System.

Figure 1 provides a comprehensive overview of the traffic monitoring and management system’s workflow. Figure 1a outlines the high-level process, beginning with input data from sensors, followed by data preprocessing to enhance video quality. The system then employs YOLO and LSTM machine learning models for training and inference, enabling vehicle detection and analysis. This results in traffic analysis and visualization, along with the generation of tracking data for monitoring vehicle movements. Figure 1b details the interaction between the video surveillance sensors and the management system. Video acquisition is followed by vehicle detection using ML-YOLO, with vehicle tracking performed using DeepSORT. The system computes traffic parameters and calculates the TPI to assess traffic conditions. Congestion classification is carried out using the K-means algorithm, and traffic trends are predicted using LSTM-Dropout. Emergency vehicles, maintenance personnel, and traffic management centers implement emergency lane control and overall traffic management strategies.

The ongoing evolution in traffic congestion prediction models, exemplified by these studies, could significantly contribute to the optimization of traffic management systems and the development of more intelligent transportation systems. These systems promise to ease motorway congestion and enhance the quality of urban life, reflecting the dynamic interplay between technology and urban planning. 

In the study of traffic congestion, there is a close relationship between traffic flow and traffic congestion. Traffic flow refers to the overall flow of vehicles on the road, while traffic congestion is a state in which vehicles travel at a reduced speed or even come to a standstill in the traffic flow. The purpose of studying traffic flow is to understand, analyze, and optimize the operation of the traffic system in order to reduce congestion, improve efficiency, and ensure safety. The analysis of traffic flow is usually based on three core elements: flow, speed, and density.

Traffic flow refers to the number of vehicles, pedestrians, and other traffic participants passing through transport infrastructure such as roads, bridges, and tunnels during a certain observation time. Its calculation formula can be expressed as Equation (1):

(1) $$Q_{t}=\frac{N}{T}$$

where $Q_{t},$ for the first t moments per unit of time, is the number of vehicles that have passed through a point, unit: veh/s; T is the length of the observation time, unit: s; and *N*, for the period of observation time, is the total number of vehicles passing through the section of the road, unit: vehicles.

Traffic speed refers to a period of time and the average speed of vehicles passing through the road. The formula can be expressed as Equation (2):

(2) $$V_{t}=\frac{1}{N}\sum_{i}^{N}V_{i}$$

where $V_{t}$, for the t section of time over the section of road, is the average speed of all vehicles traveling through and $V_{i}$ is the i vehicle’s average speed, unit: m/s.

Flow density refers to the number of vehicles within a unit length of a place at a certain time, the symbol is *K*, the formula can be expressed as Equation (3), and the unit is: veh/m. “*L*” represents the length of the road section.

(3) $$K=\frac{N}{L}$$

Traffic density is one of the key indicators describing the traffic flow situation, which reflects the traffic pressure on the road. The greater the density, the more vehicles on the road, and the higher the degree of congestion. Traffic flow is jointly determined by traffic density and traffic speed, the relationship between the three is as follows in Equation (4):

(4) $$Q_{t}=KV_{t}$$

**Figure 2:**
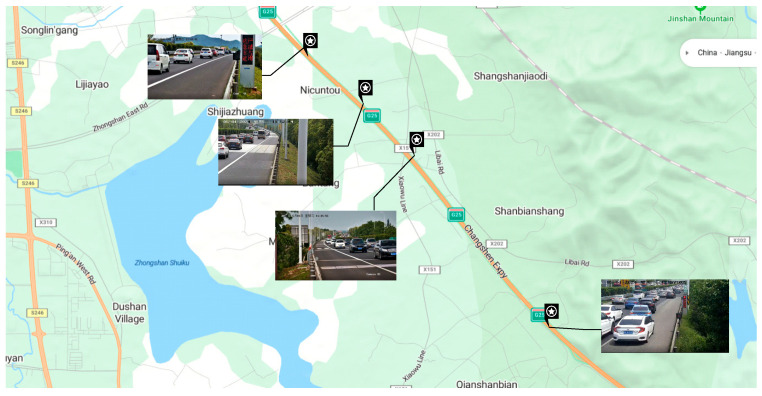
Satellite maps of monitoring sites.

**Figure 3:**
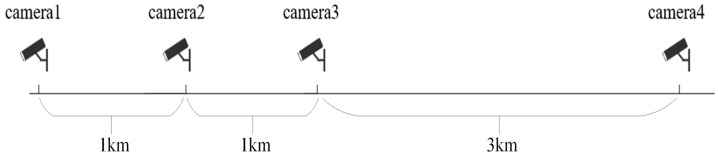
Schematic distribution of monitoring cameras.

In this research, the trend of traffic flow parameters over time is analyzed rigorously and meticulously, and the video information is transformed into more intuitive traffic flow-time cross-section information through data extraction methods. All the following video data come from four inspection cameras on the Changshen Expressway in Jiangsu Province, China, with a total length of 5 km distributed sequentially from monitoring camera 1 to monitoring camera 4. As shown in Figure 2 and Figure 3. In this study, only the congestion on the road sections from monitoring camera 3 to monitoring camera 4 is considered and their monitoring time intervals are equivalently converted into time series.

Due to the large amount of information in the video stream and the high processing difficulty, this research uses a multi-target tracking approach to process the video data from four surveillance cameras. The technique of accurately detecting and assigning unique numbers to vehicle targets in the initial frame of a video sequence, or when a new vehicle target appears, and tracking the target by matching these numbers in subsequent consecutive frames generates a complete motion trajectory of the target. The deep learning-based multi-target tracking algorithm not only relies on the appearance features extracted by the deep learning model for inter-target comparison but also incorporates the prediction of the motion trajectory, which improves the stability and accuracy of multi-target tracking.

In the complex environment of a video observation site, target detection of vehicles is challenged by a variety of natural environmental variations, especially the diversity of lighting conditions. Changes in sunlight intensity and angle at different time periods may lead to significant changes in image brightness and contrast. In addition, the complexities of roads, such as different car models and mutual occlusion between vehicles, pose additional difficulties for target detection.

**Figure 4:**
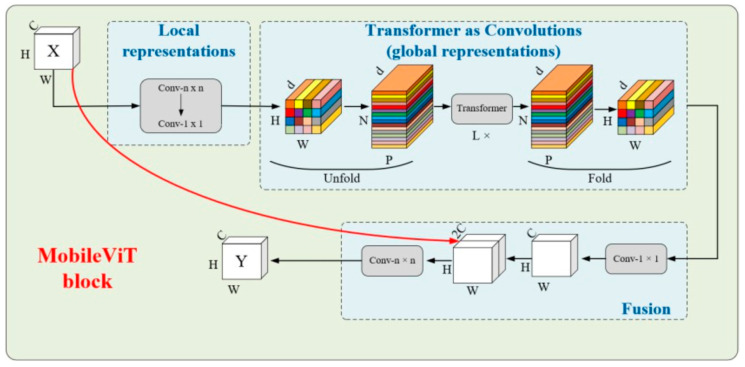
MobileViT block.

**Figure 5:**
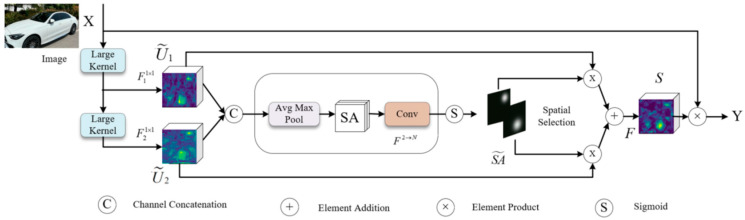
LSK module architecture.

**Figure 6:**
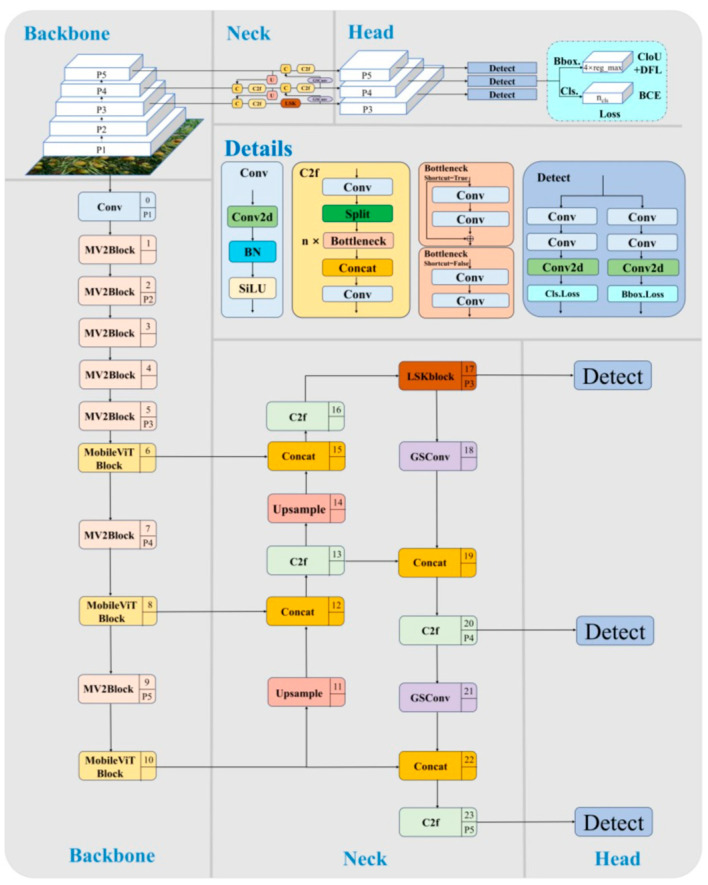
ML-YOLO model architecture.

To effectively address these challenges, this research proposes an ML-YOLO model specifically for vehicle detection in complex environments, which improves the YOLOv8n model in the following two ways. Firstly, the MobileViT block used to reconstruct the Backbone of YOLOv8n. MobileViT is a lightweight, general-purpose network that integrates the benefits of both convolutional layers and transformers. This fusion reduces the model’s size, enhancing its efficiency and making it more lightweight. The block consists of local representations obtained through convolution layers and global representations formed by transformers, which are unfolded and processed, as shown in Figure 4. The fusion of these elements effectively improves the model’s ability to handle a variety of input data while maintaining performance. Second, the LSK block uses a dynamic approach to adjust the sensing field, allowing it to better capture features from objects of various sizes and in complex backgrounds. It works by applying a spatial selection mechanism that enables the model to focus on important regions of the image, as shown in Figure 5. The interaction between the dynamic sensing field and spatial selection ensures that the model adapts to different object scales, improving detection accuracy. The LSK block effectively boosts the model’s ability to handle diverse and challenging detection tasks. Figure 6 presents the complete model architecture of ML-YOLO, detailing its structure from Backbone to Head. The architecture consists of several components, such as the MV2Block and MobileViT block within the Backbone, followed by the LSK block in the Neck for enhanced feature processing. The Head section focuses on detection, utilizing various convolution operations and loss functions to optimize the model’s performance. These blocks and their connections—ranging from data input to object detection output—are linked through convolutional layers and feature concatenation as well as spatial attention mechanisms. The architecture ensures efficient information flow across the model, maximizing detection accuracy while maintaining computational efficiency.

In order to make the training of the model more stable, firstly, a time series dataset was created by scaling the four traffic flow parameter values between 0 and 1 using MinMaxScaler. A proportion of 80% of the dataset was used for training, and the remaining 20% was used for prediction. The division was based on chronological order, ensuring that the test set contains data points from the most recent period in the time series. This method preserves the temporal dependencies in the data, which are crucial for accurate traffic prediction, as the model is trained on past data and tested on future data, reflecting real-world forecasting scenarios. Splitting the data randomly could disrupt the time-based structure, leading to unrealistic model performance.

The build model function was used to define a bi-directional LSTM-based model; the first and second layers are bi-directional LSTM and regular LSTM layers, respectively, which are responsible for extracting the time series features, and the third layer is added with a Dropout layer, which is mainly used to prevent the model from overfitting, and the output layer corresponds to the four parameters of traffic flow predicted by the model. Finally, Hyperband is used for hyperparameter optimization to ensure that the best combination of hyperparameters can be found in a short period of time.

Traffic condition evaluation criteria are an important basis for emergency lane opening decisions, which are mainly based on the degree of traffic congestion. According to YOLOv8n combined with DeepSORT target detection algorithm for video data extraction, in order to combine the speed in the video with the speed of vehicles in reality, this research maps the speed data to a new range (the minimum value is 0 m/s, the maximum value is 33 m/s), as shown in Equation (5):

(5) $$v_{\mathrm{real}\;}=\frac{v_{\mathrm{real}\;}-v_{min}}{v_{max}-v_{min}}\times \left(v_{\mathrm{newmax}\;}-v_{\mathrm{newmin}\;}\right)+v_{\mathrm{newmin}\;}$$

In the formula
$v_{\mathrm{real}\;}$ means real speed;
$v_{\min\;}$ means the minimum value of the original data;
$v_{max}$ means the maximum value of the original data;
$v_{\mathrm{newmax}\;}$ means the minimum value of the new range;
$v_{newmin}$ means the maximum value of the new range.

In this research, the TPI is used to quantify the road traffic condition, and the TPI calculation formula is shown in Equation (6):

(6) $$TPI=\frac{V_{f}}{V}\times \frac{D}{D_{max}}$$

In the formula
$\frac{V_{f}}{V}$ means the degree of deviation of the current traffic flow state from the ideal state;
$\frac{D}{D_{max}}$ means the degree of congestion on the road.

The data are extracted once every 10 s, and some of the data obtained using the target detection algorithm are shown in Table 1.

**Figure 7:**
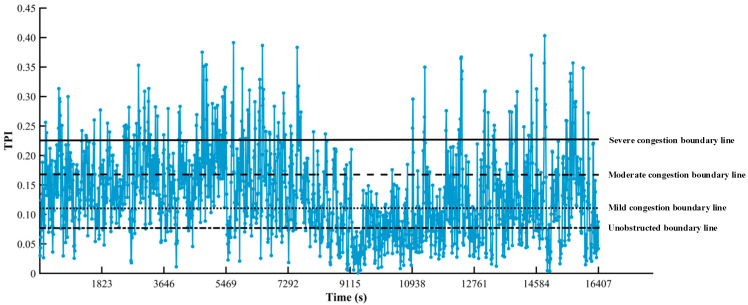
TPI-time plot for camera 3.

The traffic flow parameters detected by YOLOv8 combined with DeepSORT target detection algorithm are combined with K-means clustering algorithm to determine the TPI congestion thresholds as shown in Figure 7 and Table 2.

In order to make the decision more stable and reliable, the decision model does not only rely on the TPI value at a certain moment in time but also takes into account the trend of the TPI over a certain period of time in the future. This time period is called a time window. The purpose of choosing the time window is to observe the fluctuation of TPI over a period of time in order to avoid activating the emergency lanes too early or too late due to transient traffic flow anomalies. The time window is $T_{time}$, and according to Figure 7, the time window is chosen to be 5 min.

**Figure 8:**
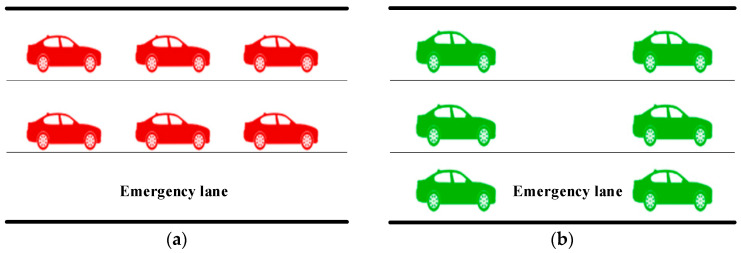
(**a**) Before using the emergency lane; (**b**) After using the emergency lane.

Utilizing the monitoring data, when congestion occurs, real-time decision-making is utilized to activate the emergency lanes, that is, the opening of the road to all three lanes, where each lane has the same capacity such that the total capacity is increased by 50%, to reduce the bottleneck effect and ease the congestion, as shown in Figure 8.

The emergency lane using the decision model is constructed based on the traffic flow parameters in the video data and the determined TPI decision threshold and combined with the traffic flow congestion prediction model based on the bi-directional LSTM-Dropout; the emergency lanes are enabled or not by comparing the predicted TPI with the TPI threshold as shown in Equation (7):

(7) $$D\left(t\right)=\left\{\begin{array}{l} 1,\frac{1}{N}\sum_{i=1}^{N}\;TPI\left(T+i\right)>{TPI}_{threshold\;}\\ 0,\frac{1}{N}\sum_{i=1}^{N}\;TPI\left(T+i\right)\leq {TPI}_{threshold\;} \end{array}\right.$$

In the formula:
D(t)=1 means the emergency lane is enabled;
D(t)=0 means the emergency lane is not activated;
TPI(T+i) means the predicted TPI value for the next minute i;
*N* means the length of the time window;
${TPI}_{\;threshold}$ means the set TPI threshold.

## 3. Results

**Figure 9:**
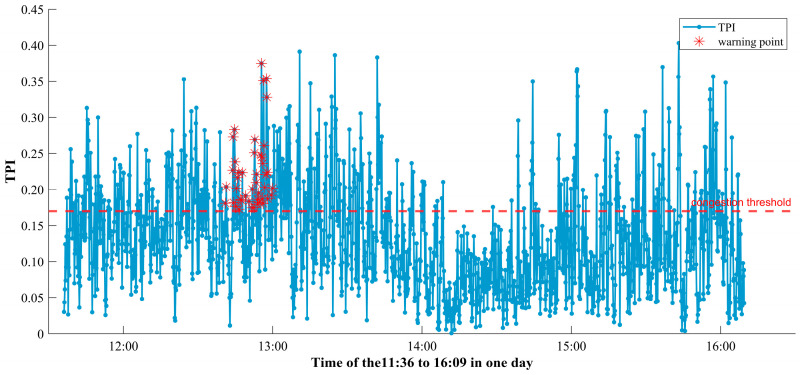
Schematic of traffic congestion warning.

According to the established two-way traffic congestion prediction model based on Long Short-Term Memory Dropout (LSTM-Dropout), the traffic congestion on the road section between the third monitoring camera and the fourth monitoring point is under a warning, and the results were shown in Figure 9.

According to the forecast result, there will be a 25 min congestion on the road section between forecast camera 3 and forecast camera 4 at 12:50 p.m.

**Figure 10:**
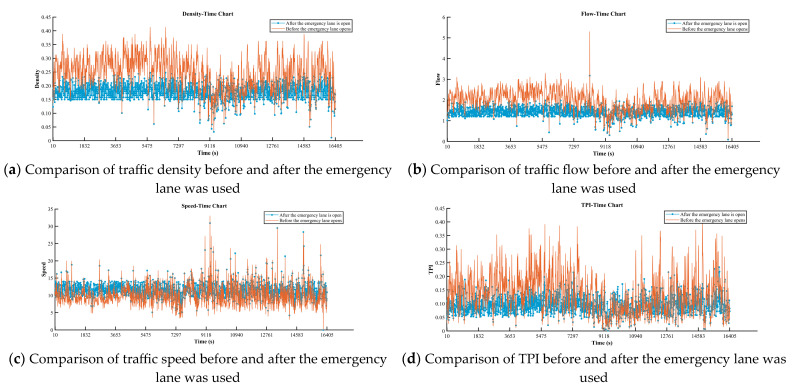
Comparison of traffic flow parameters before and after emergency lane was used.

According to the emergency lane using decision model, the emergency lane is opened when it is predicted to be persistently congested at time T, otherwise the emergency lane is closed, and the resulting traffic flow parameter pairs were shown in Figure 10.

In this research, we demonstrated that the activation of emergency lanes through a decision model led to significant improvements in traffic conditions. The Traffic Performance Index (TPI) showed a noticeable reduction, indicating improved traffic flow. At the same time, the average speed of vehicles increased, while the volume of traffic and vehicle density both decreased. These changes highlight the effectiveness of temporarily opening emergency lanes in enhancing road capacity and alleviating congestion.

## 4. Discussion

In this study, the ML-YOLO model combined with the Deep Simple Online and real-time tracking (DeepSORT) algorithm was first developed on the basis of You Only Look Once (YOLOv8n) for target detection and tracking of video data, and the traffic flow, density, speed, and Traffic Performance Index (TPI) traffic flow parameters were successfully extracted and calculated. On this basis, a traffic flow congestion prediction model and an emergency lane opening model based on two-way Long Short-Term Memory Dropout (LSTM-Dropout) were established. By substituting the data from camera 3 into the model, the warning results were obtained and the prediction results were compared with the actual situation to verify the reasonableness of the model. Table 3 showed the maximum relative error and minimum relative error values of the traffic flow parameters and calculates the Mean values and Standard Error values of the corresponding parameters.

As can be seen from the above table, the proposed model demonstrates strong performance in predicting and classifying motorway traffic congestion levels based on key parameters, including flow, density, speed, and the TPI. The evaluation metrics reveal low Mean values of 0.13 for flow, 0.04 for density, 0.11 for speed, and 0.06 for TPI, along with Standard Error values, particularly 0.01 for density and 0.02 for TPI, indicating the model’s accuracy and consistency. While speed predictions exhibited a higher Max Relative Error of 123%, attributed to inherent variability in vehicle speeds, predictions for density and TPI showed robust accuracy with Max Relative Errors of 5.67% and 7.89%, respectively. By integrating the Traffic Performance Index with the K-means clustering algorithm, the model successfully classified congestion levels into unobstructed, fairly smooth, mildly congested, moderately congested, and heavily congested categories, providing actionable insights for traffic management. For instance, when TPI exceeded 0.23, the model accurately identified heavy congestion, supporting timely interventions such as emergency lane openings to mitigate delays and enhance traffic efficiency.

Although the emergency lane management system based on the TPI demonstrates significant advantages in alleviating traffic congestion and improving emergency response efficiency, several potential challenges and limitations remain. Firstly, how to effectively and promptly warn drivers when the emergency lane is activated or closed is a key issue. Although the system uses the TPI value to predict traffic conditions, further optimization and validation are needed to ensure that drivers receive timely and accurate updates about the emergency lane status through dynamic information signs and in-vehicle notification systems. Additionally, ensuring priority access for emergency vehicles during the use of the emergency lane is another critical concern. If the emergency lane is occupied by regular traffic, it may hinder the passage of emergency vehicles, affecting the response efficiency. Therefore, integrating an emergency vehicle priority identification mechanism into the system to guarantee that emergency lanes are prioritized for urgent situations requires further system design.

Another issue that needs attention is how to quickly and effectively remove unauthorized vehicles occupying the emergency lane to ensure it is immediately available for use in emergencies. The system currently employs monitoring technologies and automated management methods for vehicle identification and clearance, but ensuring the efficiency and timeliness of this process to prevent unnecessary delays due to vehicle blockages remains a challenge in system design.

Overall, while the TPI-based emergency lane management system shows considerable potential in enhancing traffic efficiency and emergency response capability, the issues outlined above must be addressed to ensure the system can effectively handle more complex traffic situations and guarantee the efficient and precise use of emergency lanes in real-world applications.

## 5. Conclusions

In this study, we addressed the persistent issue of motorway traffic congestion by proposing an improved ML-YOLO video data extraction model combined with the Deep Simple Online and real-time tracking (DeepSORT) algorithm to classify traffic congestion levels based on real-time traffic flow parameters. The model demonstrated robust predictive accuracy across key traffic parameters, including flow, density, speed, and the Traffic Performance Index (TPI), with low Mean Absolute Error (MAE) and Mean Squared Error (MSE) values, providing a reliable theoretical basis for motorway traffic management.

1. This study introduced MobileViT and LSKb lock modules on the basis of YOLOv8n, the proposed ML-YOLO model specially used for vehicle detection in complex environments, and successfully obtained the traffic flow parameter data of a motorway.
2. This study used the TPI combined with the K-means clustering algorithm, this study effectively classified congestion into different levels based on predefined TPI thresholds. The congestion levels were categorized as “unobstructed” [0, 0.07], “fairly smooth” (0.07, 0.11], “mildly congested” (0.11, 0.17], “moderate congestion” (0.17, 0.23], and “heavy congestion” (0.23, +∞). By analyzing real-time traffic parameters such as flow, density, and speed, the clustering method enabled accurate classification of traffic conditions into these levels. This structured classification provides actionable insights for motorway traffic managers to implement targeted interventions.
3. This study introduced the LSTM-Dropout model and emergency lane using model to tackle congestion. Combined with the classified TPI threshold level, when a section of road is identified as heavily congested based on its TPI exceeding 0.17, emergency lane use can be initiated promptly, if below the threshold level, the emergency lane will be closed, to achieve intelligent emergency lane control. These models form a comprehensive framework for congestion monitoring and mitigation, contributing significantly to motorway transportation efficiency and paving the way for intelligent traffic systems in future cities.

The results of this study underscore the significant impact that intelligent traffic management systems can have on reducing congestion, optimizing traffic flow, and enhancing the efficiency of motorway transportation networks. The integration of ML-YOLO for vehicle detection, DeepSORT for real-time tracking, and advanced models like LSTM-Dropout for congestion management provides a robust framework for monitoring and mitigating congestion in a motorway. Furthermore, the proposed system holds great potential for integration with other Intelligent Transportation System (ITS) services, such as real-time traffic signal control, route optimization, and smart parking management. The fusion of these technologies can create a comprehensive, connected infrastructure that further extends the efficiency of motorway traffic management systems.

However, the methods proposed in this study have certain limitations. The model’s accuracy in real-world scenarios may be impacted by variations in environmental conditions, such as extreme weather or unexpected traffic disruptions. Additionally, the system’s performance heavily depends on the quality of real-time data inputs, which could pose challenges in environments with limited sensor coverage or unreliable data sources. Future research should focus on enhancing the robustness of these models by incorporating more diverse data sources, including IoT-based traffic sensors, vehicle-to-everything communication, and crowd-sourced traffic data. Furthermore, exploring the integration of AI-driven predictive models with real-time traffic control systems could lead to more adaptive, anticipatory traffic management strategies, further enhancing the ability to address congestion in future urban environments.

## Figures and Tables

**Table 1:** Partial sampling of flow parameters per ten seconds.

Monitoring Camera	$Q_{t}$ (veh/s)	K (veh/m)	Vehicles (veh)	$V_{t}$ (m/s)	TPI
Camera 1	0.149	0.073	11	2.04	0.11
	0.128	0.067	12	1.92	0.12
	0.155	0.090	18	1.72	0.17
Camera 2	0.218	0.063	8	3.49	0.02
	0.257	0.067	16	3.84	0.07
	0.225	0.075	20	3.00	0.13
Camera 3	0.204	0.068	16	3.00	0.04
	0.197	0.073	18	2.70	0.06
	0.210	0.079	26	2.63	0.10
Camera 4	0.416	0.072	24	5.78	0.31
	0.367	0.065	18	5.65	0.34
	0.308	0.056	17	5.50	0.32

**Table 2:** TPI congestion classification.

Congestion Level	TPI Thresholds
Unobstructed	$\left[0,0.07\right]$
Fairly smooth	$\left(0.07,\left.0.11\right]\right.$
Mildly congested	$\left(0.11,\left.0.17\right]\right.$
Moderate congestion	$\left(0.17,\left.0.23\right]\right.$
Heavy congestion	$\left(0.23,\right.$+∞)

**Table 3:** Relative error of traffic flow parameters.

Items	Maximum (%)	Minimum (%)	Mean	Standard Error
Flow	93.10	1.23	0.13	0.05
Density	5.67	0.12	0.04	0.01
Speed	123	2.34	0.11	0.04
TPI	7.89	0.23	0.06	0.02

## Data Availability

The raw data supporting the conclusions of this article will be made available by the authors on request.
